# Structural basis of antagonist selectivity in endothelin receptors

**DOI:** 10.1038/s41421-024-00705-9

**Published:** 2024-07-30

**Authors:** Junyi Hou, Shenhui Liu, Xiaodan Zhang, Guowei Tu, Lijie Wu, Yijie Zhang, Hao Yang, Xiangcheng Li, Junlin Liu, Longquan Jiang, Qiwen Tan, Fang Bai, Zhijie Liu, Changhong Miao, Tian Hua, Zhe Luo

**Affiliations:** 1 Cardiac Intensive Care Center, Zhongshan Hospital, Fudan University, Shanghai, China; 2 iHuman Institute, ShanghaiTech University, Shanghai, China; 3 School of Life Science and Technology, ShanghaiTech University, Shanghai, China; 4 Shanghai Institute for Advanced Immunochemical Studies, ShanghaiTech University, Shanghai, China; 5 Department of Anesthesiology, Zhongshan Hospital, Fudan University; Cancer Center, Zhongshan Hospital, Fudan University, Shanghai, China; 6 Shanghai Key Laboratory of Perioperative Stress and Protection, Shanghai, China; 7 Department of Critical Care Medicine, Shanghai Xuhui Central Hospital, Zhongshan Xuhui Hospital, Fudan University, Shanghai, China; 8 Shanghai Key Lab of Pulmonary Inflammation and Injury, Shanghai, China

**Keywords:** Cryoelectron microscopy, Calcium signalling

## Abstract

Endothelins and their receptors, ET_A_ and ET_B_, play vital roles in maintaining vascular homeostasis. Therapeutically targeting endothelin receptors, particularly through ET_A_ antagonists, has shown efficacy in treating pulmonary arterial hypertension (PAH) and other cardiovascular- and renal-related diseases. Here we present cryo-electron microscopy structures of ET_A_ in complex with two PAH drugs, macitentan and ambrisentan, along with zibotentan, a selective ET_A_ antagonist, respectively. Notably, a specialized anti-ET_A_ antibody facilitated the structural elucidation. These structures, together with the active-state structures of ET-1-bound ET_A_ and ET_B_, and the agonist BQ3020-bound ET_B_, in complex with G_q_, unveil the molecular basis of agonist/antagonist binding modes in endothelin receptors. Key residues that confer antagonist selectivity to endothelin receptors were identified along with the activation mechanism of ET_A_. Furthermore, our results suggest that ECL2 in ET_A_ can serve as an epitope for antibody-mediated receptor antagonism. Collectively, these insights establish a robust theoretical framework for the rational design of small-molecule drugs and antibodies with selective activity against endothelin receptors.

## Introduction

Endothelins are pivotal regulators of cardiovascular functions, essential for maintaining vascular tone and overall cardiovascular homeostasis^1^. Three endothelin peptides, namely ET-1, ET-2, and ET-3, are characterized by two unique cysteine–cysteine crosslinks^2^ and activate the endothelin receptor (ETR) subtypes ET_A_ and ET_B_^3,4^. Notably, human ET_A_ and ET_B_ receptors share 63% sequence homology^3^ but differ significantly in their ligand affinity and function. For instance, ET_A_ preferentially binds ET-1 and ET-2 over ET-3^5^, mediating strong vasoconstriction, whereas ET_B_ exhibits equal affinity for all three isoforms, primarily inducing vasorelaxation through nitric oxide and facilitating ET-1 clearance^6,7^. Consequently, ETRs are crucial targets for the treatment of cardiovascular diseases^8^.

Given the complex role of ETRs, the development of therapeutic drugs has primarily focused on vasodilatory antagonists for treating pulmonary arterial hypertension (PAH) and autoimmune diseases^9,10^. Notable selective ET_A_ antagonists or dual ET_A_/ET_B_ antagonists include bosentan, macitentan, and ambrisentan^11–15^. Additionally, ETR antagonists like sparsentan, aprocitentan, and zibotentan are currently under investigation for their potential efficacy in treating refractory hypertension and various kidney diseases^16–19^. Conversely, ET_B_ agonists are being explored for therapeutic benefits such as vasodilation and neuroprotection^20,21^. Furthermore, the development of therapeutic vaccines and monoclonal antibodies targeting ET_A_ represents an exciting frontier in PAH treatment, combining high specificity with a reduced risk of side effects^22^. Preclinical studies have shown promise in these approaches to decrease pulmonary arterial pressure^23,24^.

Structural studies on ETRs have elucidated the mechanisms of endothelin ligand recognition and activation. Extensive X-ray crystallography work has revealed the interaction patterns within the ET_B_ receptor when bound with various ligands, including ET-1, ET-3, and several antagonists^5,25–29^. Complementarily, recent cryo-electron microscopy (cryo-EM) studies on ET_A_ and ET_B_ in complex with G protein in active states have provided insights into the conserved recognition mechanisms for endogenous agonists and the selectivity for synthetic agonists between ET_A_ and ET_B_^30^. Despite these advances, the structural basis for ET_A_ antagonism remains less understood, a gap that limits the design of selective antagonists. In addition to small-molecule antagonists, a monoclonal antibody (Fab_301_) specifically targeting ET_A_ is currently in phase Ib clinical trials^23^. The specificity of antibodies may address the selectivity issues associated with small-molecule antagonists for ETRs^22,24^. However, there is currently no structural information on antibody–receptor complexes^24,31^.

This study aims to address these questions by presenting cryo-EM structures of human ET_A_ in complex with key small-molecule antagonists and Fab_301_ and by detailing the activation mechanisms of both ET_A_ and ET_B_ receptors. These molecular insights are crucial for advancing the development of therapeutics for conditions such as PAH and are part of broader efforts to enhance the specificity and efficacy of ETR-targeted drugs.

## Results

### Structure determination of antagonist-bound ET_A_ structures

**Fig. 1:**
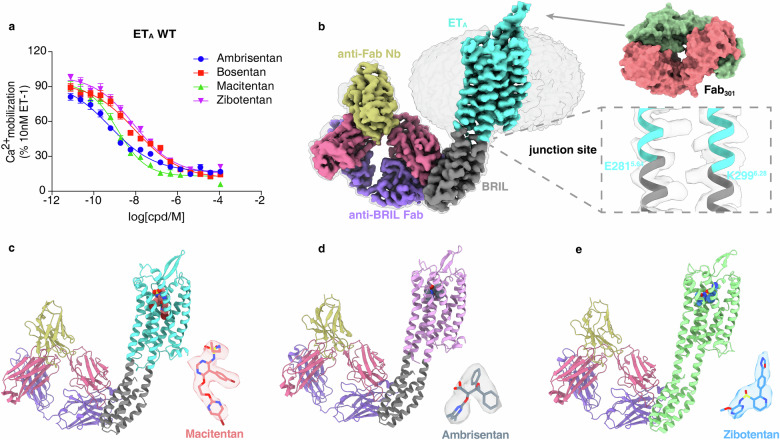
Cryo-EM structures of ET_A_ in complex with macitentan, ambrisentan, zibotentan. **a** The inhibition activity of the four different antagonists on ET_A_ in calcium mobilization assay. The IC_50_ values of macitentan, ambrisentan, zibotentan and bosentan are 1.3 nM, 0.6 nM, 8.6 nM and 9.9 nM, respectively. Data are presented as means ± SEM (*n* = 5). **b** Cryo-EM density maps of Fab_301_–ET_A_–anti-BRIL Fab-Nb complex (left panel); zoomed-in view of the junction site and the surface presentation of Fab_301_ (right panel). **c**–**e** Cartoon representation of Fab_301_–ET_A_–anti-BRIL Fab-Nb complexes with different antagonists: macitentan (**c**), ambrisentan (**d**), zibotentan (**e**). Components of ET_A_ complexes are colored as indicated. The EM density map for each ligand is shown as colored mesh.

To understand the structure basis for the antagonist binding modes in ET_A_, we analyzed three distinct compounds — macitentan, ambrisentan and zibotentan — measuring their activities on ET_A_ in calcium mobilization assay (Fig. 1a; Supplementary Fig. S1). Determining the structure of inactive-state G protein-coupled receptors (GPCRs) via cryo-EM is inherently challenging due to the absence of the heterotrimeric G protein, which is essential for particle alignment in active GPCR structure determination. Here, we developed an optimized strategy to solve the inactive ET_A_ structures. Initially, to overcome the low surface expression of ET_A_, endoglucanase H was fused to the N-terminus of ET_A_, along with N- and C-terminal truncations (Materials and methods). Then, to compensate for the absence of the G protein, a thermostabilized apocytochrome b562 RIL (BRIL)^32^ protein was fused between TM5 and TM6, replacing ET_A_’s third intracellular loop (ICL3) (Supplementary Fig. S1a). The design of the BRIL fusion sites for uninterrupted helicity was informed by AlphaFold2^33^ predictions.

Subsequent stabilization of the modified ET_A_ construct involved the introduction of a BRIL-binding Fab and a nanobody (Nb) that reinforces the hinge region, as previously described^34,35^. Despite these modifications, we encountered stability and orientation preferences during cryo-EM grid preparation. To address these issues, we introduced an ET_A_-specific antibody, Fab_301_^23^, which not only conferred additional stability to the complex but also exhibited antagonistic effects against ET-1-induced ET_A_ signaling. These strategic modifications enabled us to determine the cryo-EM structures of Fab_301_-bound ET_A_ in complex with macitentan, ambrisentan and zibotentan, at nominal resolutions of 3.1 Å, 3.2 Å and 3.2 Å, respectively (Fig. 1b−e; Supplementary Fig. S2 and Table S1). The high-resolution cryo-EM maps provided detailed electron density in the orthosteric ligand-binding pocket, enabling accurate placement of each antagonist (Supplementary Fig. S2). It is noteworthy that the interaction interface between Fab_301_ and ET_A_ is quite limited, and within the cryo-EM particles, Fab_301_ appears to exhibit a degree of movement.

Comparative analysis of the macitentan–ET_A_, ambrisentan–ET_A_ and zibotentan–ET_A_ complexes reveals a conserved conformation among the three structures, with C_α_ root mean square deviation (RMSD) values between 0.7 Å and 1.0 Å (Supplementary Fig. S3a, b). Compared to the crystal structures of antagonist-bound ET_B_, both receptors adopt a similar inactive conformation, with an RMSD of 1.5 Å (Supplementary Fig. S3c).

### Binding of macitentan to ET_A_

**Fig. 2:**
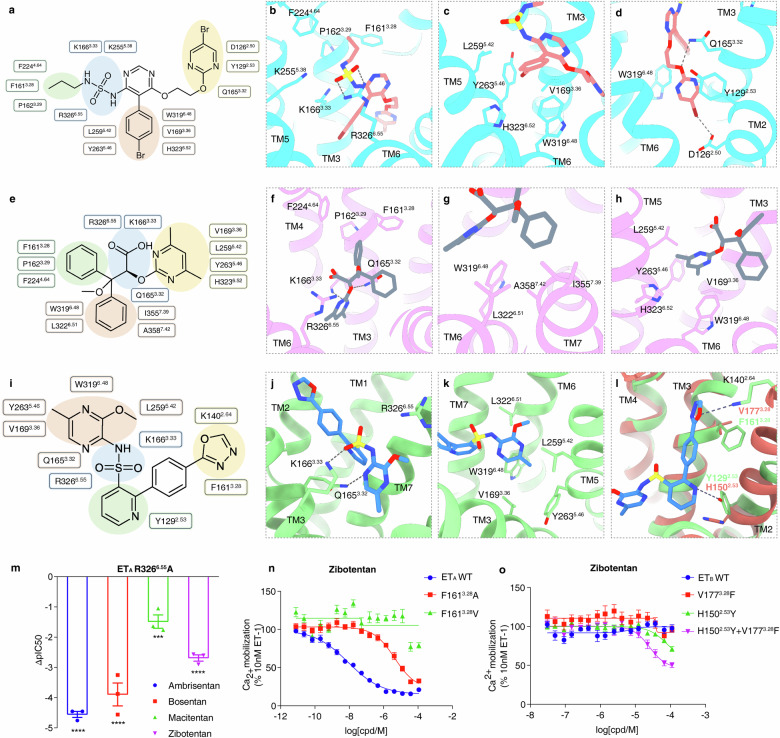
Characterization of macitentan, ambrisentan, and zibotentan binding modes in ET_A_. **a**–**d** Schematic of macitentan’s interactions with key residues in ET_A_. **e**–**h** Schematic of ambrisentan’s interactions with key residues in ET_A_. **i**–**l** Schematic of zibotentan’s interactions with key residues in ET_A_. The selectivity of zibotentan towards ET_A_ may be attributed to Y129^2.53^ in ET_A_ but H150^2.53^ in ET_B_ (**l**). **m**–**o** The antagonistic effects of four different antagonists on the ET_A_ R326^6.55^A variant (**m**); effects of mutations in ET_A_ (**n**) and ET_B_ (**o**) on zibotentan’s antagonistic activity. ΔpIC_50_ represents the difference in pIC_50_ values between the wild type (WT) and the mutants of ET_A_. Data are presented as means ± SEM (*n* ≥ 3). Hydrogen bonds are highlighted with gray dashed lines.

Macitentan, designed as an ET_A_-selective antagonist, is a derivative of the dual-acting antagonist bosentan. In our calcium mobilization assay, macitentan shows a high affinity to ET_A_, with an IC_50_ of 1.3 nM (Fig. 1a; Supplementary Table S2), contrasting to the lower affinity to ET_B_ at 14.5 μM (Supplementary Fig. S4b), which is also consistent with the previous research results^36^. Structurally, macitentan retains the pyrimidine core characteristic of bosentan but distinguishes itself with a sulfonamide substitution at the fourth position^37^ (Supplementary Fig. S1b, c). The binding of macitentan within the ET_A_ pocket involves several distinct interactions (Fig. 2a). The sulfonamide moiety in macitentan establishes a hydrogen bond with R326^6.55^ (Fig. 2b), and forms ionic interactions with residues K166^3.33^, K255^5.38^ and R326^6.55^ from ET_A_ (Fig. 2b). The bromophenyl group is embedded in the receptor’s hydrophobic core, forming hydrophobic interactions with residues W319^6.48^, V169^3.36^, Y263^5.46^, H323^6.52^, and L259^5.42^ from TMs 3, 5, and 6 (Fig. 2c), and is further stabilized by a cation–π interaction with K166^3.33^ (Fig. 2b). The 2-(5-bromopyrimidin-2-yl)oxyethoxy component, linked to the sixth position of the pyrimidine, allows the oxygen atom in the bromopyrimidine to form a hydrogen bond with Q165^3.32^ (Fig. 2d). Importantly, this moiety inserts deeper into the orthosteric pocket than the similar groups in bosentan or K-8794 in ET_B_, positioned to form a halogen bond with D126^2.50^ (Fig. 2d). Furthermore, bromopyrimidine engages in π–π interactions with Y129^2.53^ and the “toggle switch” residue W319^6.48^ (Fig. 2d). The ethyl tail attached to the sulfonamide moiety is nestled within a hydrophobic pocket formed by F161^3.28^, P162^3.29^, and F224^4.64^ from TM3 and TM4 of ET_A_. Prior research has demonstrated that substituting the sulfonamide moiety with sulfamide significantly boosts ETR antagonists’ receptor affinity^11^. Our structural analysis reveals that the addition of the -NH- group in macitentan contributes to a more stable electrostatic network with K166^3.33^ and K255^5.38^, enhancing macitentan’s binding affinity (Fig. 2b). Notably, the R326^6.55^A mutation showed minimal influence on macitentan’s activity on ET_A_, aligning with macitentan’s stronger electrostatic network at this location in ET_A_ (Fig. 2m).

### Binding of ambrisentan to ET_A_

Ambrisentan, a propionic acid derivative discovered through high-throughput screening, differentiates itself from macitentan by featuring a carboxylic acid group instead of a sulfonamide moiety^15^ (Supplementary Fig. S1d). Despite its smaller molecular weight relative to macitentan and zibotentan, ambrisentan exhibits a remarkably high affinity to ET_A_, befitting its role as an ET_A_-selective antagonist (Fig. 1a). The structural elucidation of its binding to ET_A_ uncovers essential interactions. The carboxylic acid group forms ionic interactions with the positively charged residues K166^3.33^ and R326^6.55^ (Fig. 2f), while a network of hydrogen bonds with residues R326^6.55^, K166^3.33^, and Q165^3.32^ firmly anchors ambrisentan in the binding site (Fig. 2e, f). Moreover, the hydrophobic interaction of one benzene ring from the symmetric pair in ambrisentan with residues F161^3.28^, P162^3.29^, and F224^4.64^ creates a snug fit within a subpocket (Fig. 2f). The counterpart benzene ring engages with residues L322^6.51^, W319^6.48^, I355^7.39^, and A358^7.42^ spanning TM6 and TM7 (Fig. 2g). Additionally, the methyl groups on the pyrimidine ring of ambrisentan adapt to the hydrophobic cores formed by residues V169^3.36^, L259^5.42^, Y263^5.46^, and H323^6.52^ from TMs 3, 5 and 6 (Fig. 2h), further stabilizing the antagonist binding within the receptor. Furthermore, our molecular dynamics (MD) simulations indicate the stable binding of ambrisentan within the pocket (Supplementary Fig. S5).

### Binding of zibotentan to ET_A_

Zibotentan, a sulfonamide-based compound, exhibits selective antagonism towards ET_A_ with an IC_50_ of 8.6 nM in our calcium mobilization assay (Fig. 1a). Structurally distinguished by a central pyridine ring flanked by two bulky substituents (Fig. 2i), zibotentan adopts a chair-like conformation within the orthosteric binding pocket, contrasting with the spatial arrangement of macitentan (Supplementary Fig. S1c, e). The sulfonamide group forms electrostatic interactions with Q165^3.32^, K166^3.33^ and R326^6.55^ (Fig. 2j), whereas the pyrazinyl rings, modified with methoxy and methyl groups, engage in hydrophobic contacts with residues around W319^6.48^ from TMs 3, 5 6 (Fig. 2k). On the opposite end, the 1,3,4-oxadiazol-2-yl segment of zibotentan extends towards ECL2, forming a π–π interaction with F161^3.28^ and a hydrogen bond with K140^2.64^ (Fig. 2l). Furthermore, Y129^2.53^ appears to participate in polar interactions or hydrogen bonds with the pyridine ring’s nitrogen (Fig. 2l).

Subsequent structural investigations suggest that zibotentan’s selectivity for ET_A_ may be significantly influenced by the residue F161^3.28^, which is a valine (V177^3.28^) in ET_B_. This phenylalanine F161^3.28^ acts as a “tray” that stabilizes one end of the zibotentan molecule (Fig. 2l). Concordantly, mutation of F161^3.28^A or F161^3.28^V significantly reduces zibotentan’s activity on ET_A_ (Fig. 2n). Additionally, the interaction with Y129^2.53^ is also crucial; its alteration to phenylalanine or histidine (Y129^2.53^F or Y129^2.53^H) markedly reduces zibotentan’s activity on ET_A_, highlighting the importance of this residue for antagonist specificity (Supplementary Fig. S4c−e). Intriguingly, introducing corresponding mutations into ET_B_ (V177^3.28^F and H150^2.53^Y) partially impairs the activity of zibotentan on ET_B_, which underscores the critical role of these residues in the determination of antagonist selectivity in ET_A_ or ET_B_ subtype (Fig. 2o).

### Structure basis of antagonist selectivity in ET_A_ and ET_B_

**Fig. 3:**
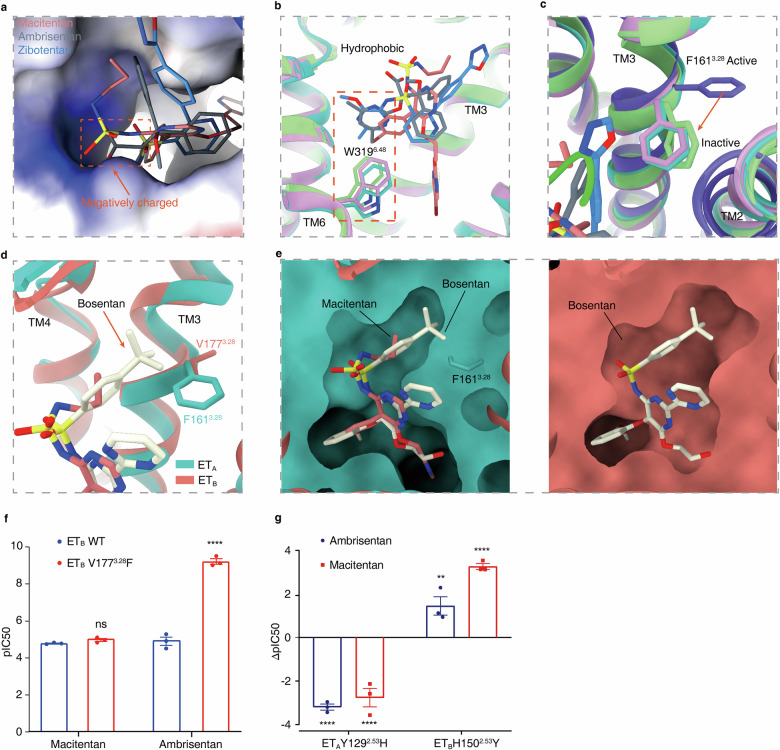
Structure basis of antagonist selectivity in ET_A_ and ET_B_. **a** The sulfonylamide in macitentan and zibotentan, along with the carboxylic acid group in ambrisentan, engage with the positively charged area (blue surface) in ET_A_’s orthosteric pocket. **b** Three antagonists hinder the inward movement of W319^6.48^’s side chain through analogous functional groups at the similar positions. **c** The conformation of F161^3.28^ in different states. **d** In ET_B_’s corresponding position, V177^3.28^, offers a larger antagonist-binding pocket. **e** Different spatial accommodation of antagonists in ET_A_ and ET_B_. The bulky hydrophobic group (4-t-butylphenyl) of bosentan is better adapted to ET_B_, whereas the smaller hydrophobic group of macitentan shows a preference for ET_A_. **f** Effects of V177^3.28^F in ET_B_ on the antagonistic activities of ambrisentan and macitentan (*n* = 3). **g** Difference in pIC_50_ values between ET_A_ and ET_B_ variants and WT in antagonist experiments. Data are presented as means ± SEM (*n* ≥ 3). All data were analyzed by one-way ANOVA by Dunnett’s multiple test compared with WT. For mutants, *P < 0.05, **P < 0.01, ***P < 0.001 and ****P < 0.0001 were considered statistically significant.

Structural elucidation of ET_A_ in complex with various antagonists, each featuring distinct scaffold architectures, advances our understanding of the antagonist binding modes in ET_A_ and delineates commonalities critical for their antagonistic function. An important aspect of their activity is the interaction with the positively charged region in ET_A_’s orthosteric pocket. Functionally important groups, such as the sulfonylamide in bosentan, macitentan, and zibotentan, along with the carboxylic acid group in ambrisentan, are essential. These functional groups occupy the position corresponding to W21, the C-terminal end of the endogenous agonist ET-1 (Fig. 3a), a key engagement site with ET_A_^30^. Another shared trait of these antagonists is their interaction in close proximity to TM5 and TM6, which helps stabilize ET_A_ conformation in this region (Fig. 3b). The three antagonist-bound structures display a hydrophobic moiety in this vicinity, forming hydrophobic contacts with residues from TMs 3, 5 and 6 (Fig. 3b). These groups also hinder the inward movement of the side chain of W319^6.48^ through hydrophobic or π–π interactions (Fig. 3b). Mutagenesis data, such as L259^5.42^A, Y263^5.46^A or L322^6.51^A, further confirm the critical role of these hydrophobic contacts in sustaining antagonist efficacy (Supplementary Fig. S4f−h).

Regarding the selectivity differences, we observed that, in ET_A_, F161^3.28^ rotates inwards to the orthosteric pocket in the antagonist-bound state, which results in a compact antagonist-binding pocket in ET_A_ (Fig. 3c). In contrast, in the corresponding site in ET_B_, a smaller V177^3.28^ yields a more expansive antagonist-binding pocket (Fig. 3d). This spatial variation accounts for the more effective accommodation of the larger bulky hydrophobic group (4-t-butylphenyl) of bosentan within ET_B_, while the smaller hydrophobic groups of macitentan and ambrisentan show a preference for ET_A_ (Fig. 3e, f). Regarding zibotentan’s ET_A_ selectivity, as previously discussed, this may be attributed to the different residue Y129^2.53^ in ET_A_ compared to H150^2.53^ in ET_B_ (Figs. 2l, 3g). Together, Y129^2.53^ and F161^3.28^ in ET_A_ may partially account for the observed selectivity of antagonists toward ET_B_.

### Active and inactive conformation features of ETRs

**Fig. 4:**
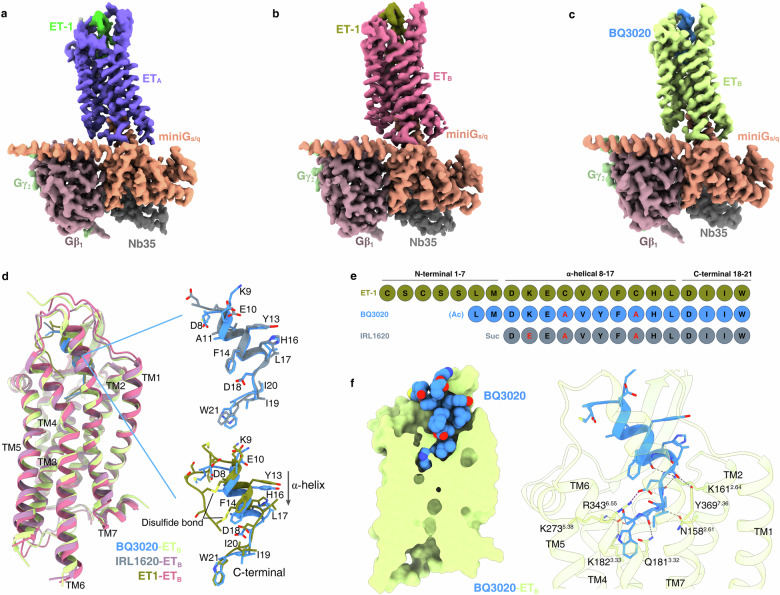
Cryo-EM structures of BQ3020–ET_B_–miniG_s/q_–Nb35, ET1–ET_B_–miniG_s/q_–Nb35, and ET1–ET_A_–miniG_s/q_–Nb35 complexes. **a**–**c** Cryo-EM density maps of ET1–ET_A_–miniG_s/q_–Nb35 (**a**), ET1–ET_B_–miniG_s/q_–Nb35 (**b**), and BQ3020–ET_B_–miniG_s/q_–Nb35 (**c**) complexes. **d** Structural comparison of ET_B_ bound to BQ3020, IRL1620, and ET-1. Conformational comparison between BQ3020 and IRL1620 in the binding pocket (upper right), and between BQ3020 and ET-1 (bottom right). The arrow indicates that the α-helix in the binding pocket shifts slightly downward. **e** Sequence alignment of peptides including ET-1, BQ3020 and IRL1620. **f** Cross-section of the BQ3020-binding pocket in ET_B_. BQ3020 is displayed as spheres (left panel). Detailed interactions between BQ3020 and ET_B_ are shown (right panel).

To delve deeper into the activation mechanisms of ETRs, we determined the structures of ET_A_ and ET_B_ in their active states, at global resolutions of 3.3 Å for ET-1-bound ET_A_–miniG_s/q_–Nb35 complex and 3.2 Å for ET_B_–miniG_s/q_–Nb35 complex (Fig. 4a, b; Supplementary Fig. S6 and Table S1). In addition, a structure of ET_B_ complexed with the selective agonist BQ3020^38^, was determined at a resolution of 3.0 Å (Fig. 4c; Supplementary Fig. S6a and Table S1). The overall conformations of ET-1-bound ET_A_–miniG_s/q_ and ET_B_–miniG_s/q_ complex structures, including the ET-1 binding poses, are consistent with previously reported ETR structures^30^ (Supplementary Fig. S7a, b). Notably, the binding pose of BQ3020 in ET_B_ closely resembles that of ET-1, with a C_α_ RMSD of 0.8 Å for the receptor (Fig. 4d, f). BQ3020, which differs from the ET_B_-selective agonist IRL1620 by a single residue (Fig. 4e), exhibits an overall structural similarity, as the BQ3020–ET_B_ and IRL1620–ET_B_ complex structures display an RMSD of 0.9 Å. This indicates the aligned positioning of the agonists within the binding pocket (Fig. 4d). In the BQ3020–ET_B_ complex, BQ3020’s C-terminal configuration closely resembles that of ET-1, albeit with a slightly downward shift in the α-helix within the binding pocket, due to the absence of disulfide-bond constraints (Fig. 4d).

Additionally, the mutation W146^ECL1^A in ET_A_ does not significantly affect receptor activation, whereas the analogous mutation W167^ECL1^A in ET_B_ results in a substantial reduction in ET_B_ activation (Supplementary Fig. S7c and Table S3). This suggests that ET_B_ requires interactions with larger hydrophobic groups at this site for activation. Within ET_B_’s ECL1, F169^ECL1^ is posited to engage in a π–π interaction with W167^ECL1^ (Supplementary Fig. S7d), a key conformation for receptor function. Alanine mutation of F169^ECL1^ hampers ET-1’s capability to activate ET_B_ (Supplementary Fig. S7c).

**Fig. 5:**
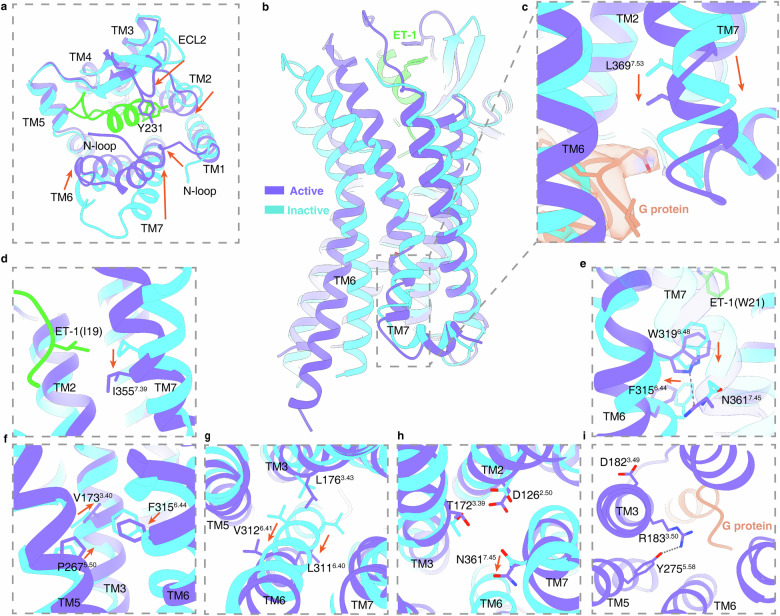
Structural comparison of Fab_301_–macitentan–ET_A_–anti-BRIL Fab-Nb and ET1–ET_A_–miniG_s/q_–Nb35 complexes. The purple cartoon represents ET1-bound ET_A_, whereas cyan cartoon represents macitentan-bound ET_A_. **a** Conformational changes in loop regions and helical rearrangement during ET_A_ activation (top view). **b**, **c** Structural comparison reveals the outward extension of TM6 (**b**) and the movement of the cytoplasmic portion of TM7 (**c**). **d** The downward movement of the side chain of I19 in ET-1-bound ET_A_ structure. **e**–**i** Conformational changes of key motifs related to ET_A_ activation. NPxxY (**c**), PIF motif (**e**, **f**), DRY (**i**).

Employing the Residue–Residue Contact Score (RRCS) tool^39^, we analyzed structures bound by three antagonists, confirming that the residue contacts are characteristics of the inactive-state class A GPCRs (Supplementary Fig. S8). A comparative analysis of the macitentan-bound ET_A_ structure against the ET-1-bound ET_A_ structure reveals significant conformational changes when ET_A_ transits from the inactive to active states. ET-1 binding promotes an inward movement of the extracellular portions of TM2, TM6 and TM7, resulting in a more compact receptor core (Fig. 5a). Concurrently, ECL2 moves inwards substantially, acting as a “lid” that secures ET-1 in place, facilitated by a π–π interaction between ET-1’s Y13 and Y231^ECL2^ in ET_A_ (Fig. 5a). However, we cannot exclude the influence of Fab_301_ on the conformational changes of ECL2 when compared to the state bound alone by the small-molecular antagonist. The EM density map allowed the modeling of extended N-terminal residues, revealing a tighter packing with ECL2 and ECL3 in the ET-1-bound ET_A_ structure compared to the macitentan-bound state (Fig. 5a). These structural rearrangements lead to a more compact orthosteric pocket for ET-1 interaction. The characteristic outward displacement of the cytoplasmic part of TM6 by 9.7 Å in the ET-1-bound ET_A_–miniG_s/q_ structure, reflects the conformational changes, indicative of class A GPCR activation (Fig. 5b). Moreover, the intracellular portion of TM7 undergoes a displacement of ~2.9 Å (measured by the C_α_ atom of L369^7.53^), in the active state (Fig. 5c).

### Mechanism for ET_A_ activation

The antagonist-bound ET_A_ structures illuminate the underlying activation mechanism. Upon ET-1 binding, the side chain of I19 residue exerts pressure on I355^7.39^, prompting a downward shift of this residue and the associated intracellular half of TM7 (Fig. 5d). Concurrently, ET-1’s W21 residue engages with the “toggle switch” residue W319^6.48^ (Fig. 5e). This interaction induces a downward rotation of W319^6.48^, facilitating a hydrogen bond formation between its nitrogen atom and N361^7.45^ (Fig. 5e). The downward motions of W319^6.48^ and N361^7.45^ promote an inward-to-outward rotation of F315^6.44^ within the P^5.50^I/V^3.40^F^6.44^ motif (Fig. 5e, f), triggering the outward movement of TM6 at the cytoplasmic end (Fig. 5e). These sequential events are accompanied by the disruption of interactions between L176^3.43^, L311^6.40^ and V312^6.41^, which results in the release of the stacking of TM3 and TM6, further promoting the outward swing of TM6 (Fig. 5g). Additionally, the downward movement of N361^7.45^ leads to the collapse of the Na^+^ pocket, previously stabilized by D126^2.50^, T172^3.39^, and N361^7.45^ in the inactive state, and triggers subsequent rearrangements between TM7, TM3 and TM2 (Fig. 5h).

Regarding the highly conserved N^7.49^P^7.50^xxY^7.53^ motif in class A GPCRs, ET_A_ and ET_B_ feature a variant of N^7.49^P^7.50^xxL^7.53^Y^7.54^, wherein Y^7.53^ is replaced by L^7.53^. During ET_A_ activation, L179^3.46^ does not engage in a polar inter-helix interaction, which permits L369^7.53^ to move downward, enhancing its coupling with the C-terminus of the G protein’s α5 helix (Fig. 5c). Concerning the D^3.49^R^3.50^Y^3.51^ motif, ET_A_ activation disrupts the conserved ion lock between D182^3.49^ and R183^3.50^. The released R183^3.50^ then forms a polar interaction with Y275^5.58^ and establishes interactions with the α5 helix of the G protein, anchoring the receptor in its active state (Fig. 5i).

### Insights into antibody design to antagonize ET_A_

**Fig. 6:**
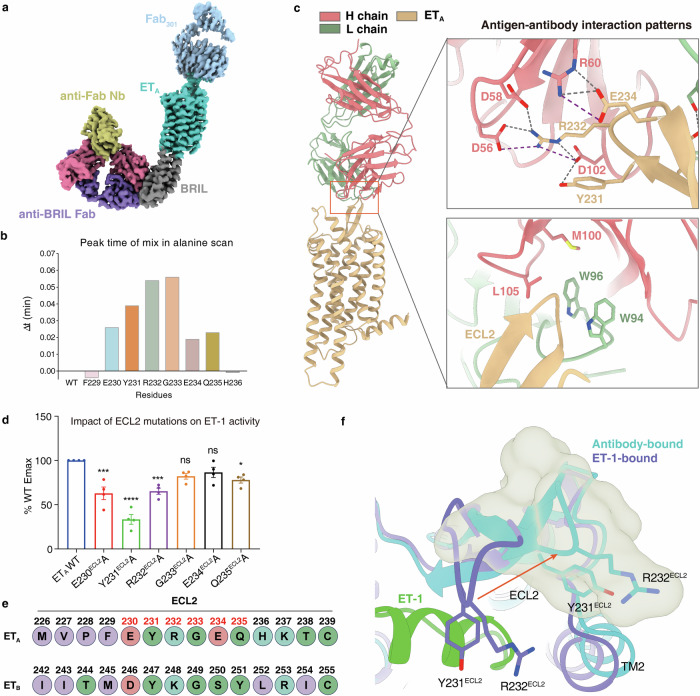
Binding interface between Fab_301_ and ET_A_. **a** Cryo-EM density maps showing the Fab_301_ binding to the ECL2 region of ET_A_. **b** Differences between the retention time of each mutant–Fab_301_ complex and the WT–Fab_301_ complex. The letter “Δt” represents the retention time of the mutant sample minus the retention time of the WT sample. **c** The Fab_301_–ET_A_ complex predicted by AlphaFold2 multimer was modeled, which fits well with the experimental electron density of Fab_301._ Zoomed-in view of the antibody-binding interface is shown (right panel). **d** Response of ET-1 on WT ET_A_ and ET_A_ mutants in ECL2. Data are presented as means ± SEM (*n* = 4). All data were analyzed by one-way ANOVA by Dunnett’s multiple test compared with WT. *P < 0.05, **P < 0.01, ***P < 0.001 and ****P < 0.0001 were considered statistically significant. **e** Sequence alignment of the ECL2 regions between ET_A_ and ET_B_. Amino acids are classified according to their properties. **f** Conformational change of ECL2 between ET-1-bound ET_A_ and Fab_301_-bound ET_A_. The gray area represents the Fab_301_ density, and the arrow indicates that the ECL2 expands outward in the inactive structure.

In our structural analysis of the antagonists-bound ET_A_, the employed Fab_301_ shows antagonistic effect and facilitates the structure determination. The EM density maps clearly reveal the binding of Fab_301_ to ET_A_’s ECL2 region (Fig. 6a). However, due to the dynamic nature of the interaction between Fab_301_ and ET_A_’s ECL2, pinpointing their precise binding interface is challenging. We addressed this by using AlphaFold2 multimer^33,40^ prediction to model the Fab_301_–ET_A_ complex interface (Supplementary Fig. S9a). Ultimately the epitope of Fab_301_ was roughly located based on the fitting of the predicted model with the experimental EM densities (Fig. 6c).

To pinpoint the exact ECL2 residues involved in binding, we performed an extensive alanine scanning mutagenesis. Our size exclusion chromatography analysis identified that the region spanning residues 230^ECL2^–235^ECL2^ in ET_A_, and in particularly, residues R232^ECL2^ and G233^ECL2^, are crucial for Fab_301_ binding. Mutations of R232^ECL2^A or G233^ECL2^A resulted in a near-complete loss of Fab_301_’s binding to ET_A_ (Fig. 6b; Supplementary Fig. S10). These results complement our predicted binding mode, where R232^ECL2^ of ET_A_ forms an important polar interaction network with Fab_301_ (Fig. 6c). Moreover, the hydrophobic interactions at this interface are crucial for antibody binding, as confirmed by our calcium mobilization assay on Fab_301_ (Supplementary Fig. S9b). Within this model, E230^ECL2^ establishes hydrogen bonds with the CDRL1 and CDRL2 regions of Fab_301_, whereas Y231^ECL2^ and E234^ECL2^, together with R232^ECL2^, participate in a complex hydrogen bond network within the CDRH3 region of Fab_301_ (Fig. 6c). Alanine mutations on E230^ECL2^, Y231^ECL2^, and R232^ECL2^ also affected ET-1’s activity on ET_A_. Notably, Y231^ECL2^ mutation showed the most significant effect in calcium mobilization assay (Fig. 6d). These data underscore the essential role of ECL2 in the recognition of ligands and Fab_301_ by ET_A_.

Comparative structural analysis of ET_A_ bound by ET-1 and Fab_301_ illustrates how ECL2 residues E230^ECL2^, Y231^ECL2^, and R232^ECL2^, which directly interact with ET-1, undergo a conformational change upon Fab_301_ binding. This change fosters a polar interaction network with Fab_301_, effectively inhibiting ET-1 binding to ET_A_ (Fig. 6f). Additionally, an ECL2 sequence alignment between ET_A_ and ET_B_ uncovers the basis for Fab_301_’s selectivity for ET_A_ over ET_B_ (Fig. 6e). The distinct sequence variation, specifically at residues E230–Q235 of ET_A_, is pronounced between the two receptors. This divergence provides a valuable template for the design of selective antibodies targeting the ETR subtypes.

## Discussion

The structural elucidation of antagonist-bound ET_A_ in this study provides critical insights into the mechanisms underpinning receptor selectivity and activity modulation. The cryo-EM structures of ET_A_ in complex with macitentan, ambrisentan, and zibotentan underscore the intricate molecular interactions that govern antagonist binding.

The analysis of antagonist-bound ET_A_ structures reveals conserved features aligning with the inactive state observed in antagonist-bound ET_B_ structures, suggesting common structural themes in ETR antagonism. Macitentan’s high affinity to ET_A_ is attributed to several key interactions, including hydrogen bonds and ionic interactions within the orthosteric pocket. The unique sulfonamide moiety enhances these interactions, resulting in a stable electrostatic network crucial for high binding affinity. Similarly, ambrisentan’s high affinity and selectivity are due to its carboxylic acid group forming robust ionic and hydrogen bonds, coupled with hydrophobic interactions that create a snug fit within the binding pocket. Zibotentan’s unique chair-like conformation and its interactions with residues around TM3, TM5, and TM6 further highlight the structural adaptations that facilitate selective binding.

The selectivity of antagonists for ET_A_ over ET_B_ is primarily influenced by specific residues within the binding pocket. F161^3.28^ in ET_A_, which rotates inward in the antagonist-bound state, creates a compact binding pocket that favors smaller hydrophobic groups, whereas the corresponding V177^3.28^ in ET_B_ accommodates larger groups. The residue Y129^2.53^ in ET_A_, compared to H150^2.53^ in ET_B_, further contributes to this selectivity by engaging in critical polar interactions.

The antagonistic effect of Fab_301_ and its ability to stabilize the ET_A_ structure were utilized to gain further insights into antibody–ET_A_ interactions. The binding of Fab_301_ to the ECL2 region of ET_A_ involves critical residues, notably R232^ECL2^ and G233^ECL2^, essential for Fab_301_’s binding affinity. The detailed mapping of the Fab_301_–ET_A_ interface provides a template for designing selective antibodies that target specific ETR subtypes.

This study enhances our understanding of the structural basis for antagonist selectivity and activation mechanisms of ETRs. The high-resolution structures of antagonist-bound ET_A_ reveal conserved features essential for selective binding and receptor stabilization. Key residues within the orthosteric pocket play crucial roles in determining antagonist affinity and selectivity, offering valuable insights into the design of selective therapeutic agents.

## Materials and methods

### Cell lines

*Spodoptera frugiperda* (*Sf*9, expression systems) and *Trichoplusia ni* (High Five, Thermo Fisher Scientific) cells were gown in ESF medium at 27 °C and 120 rpm. CHO-K1 cells were cultured in Ham’s F-12K (Kaighn’s) Medium (Gibco-Thermo Fisher Scientific) supplemented with 10% (v/v) fetal bovine serum (FBS; Gibco-Thermo Fisher Scientific), and 100 U/mL Penicillin-Streptomycin (Gibco-Thermo Fisher Scientific) in a humidified incubator at 37 °C with 5% CO_2_.

### ET_A_ and ET_B_ construct design

For the complex structures of G protein-coupled ET_A_ and ET_B_, the human *ET*_*A*_ or *ET*_*B*_ genes were cloned into pFastBac1 vector. This vector was modified to include a hemagglutinin (HA) signal peptide, a Flag tag at the N-terminus of the receptor, an HRV3C protease recognition site, and a 10× His tag at the C-terminus. To improve the protein yield of ET_A_, we fused an endoglucanase H (PDB: 2CIT) at the N-terminus of ET_A_, simultaneously truncating residues 1–49 at the N-terminus and residues 406–427 at the C-terminus. Similarly, for ET_B_, we truncated N-terminal residues 1–66 and C-terminal residues 407–442 and fused a sialidase H (PDB: 2VK5) to the N-terminus.

For the antagonist-bound ET_A_ structure determination, the cytochrome b562RIL (BRIL) protein was inserted into the ICL3, specifically replacing residues 282–298 of ET_A_. Additionally, we appended sequences ARRQL and ERARSTL from A_2A_ adenosine receptor to the N-terminus and C-terminus of BRIL, respectively^41^. The truncations for ET_A_ were consistent with those described above.

### ET_A_–miniG_s/q_–Nb35 and ET_B_–miniG_s/q_–Nb35 complex formation and purification

The ET_A_–miniG_s/q_–Nb35 and ET_B_–miniG_s/q_–Nb35 complexes were assembled in vitro using modified ET_A_ and ET_B_, miniGα_s/q_, Gβ_1_γ_2_ and Nb35. The expression and purification of miniGα_s/q_, Gβ_1_γ_2_ and Nb35 were performed as previously described^42^. ET_A_ and ET_B_ were expressed in *Sf*9 insect cells using the Bac-to-Bac baculovirus expression system (Invitrogen). The cells were cultured at 27 °C and collected 48 h after infection. Subsequently, 2 L cell pellets were sequentially washed and centrifuged with hypotonic buffer (10 mM HEPES, pH 7.4, 20 mM KCl, 10 mM MgCl_2_, EDTA-free protease inhibitor cocktail tablets), followed by a high osmotic buffer (10 mM HEPES, pH 7.4, 1 M NaCl. 20 mM KCl, 10 mM MgCl_2_, EDTA-free protease inhibitor cocktail tablets). The purified membrane was solubilized with a buffer (50 mM HEPES, pH 7.4, 100 mM NaCl, 1% (w/v) lauryl maltose neopentyl glycol (LMNG, Anatrace), 0.2% (w/v) cholesterol hemisucinate (CHS, Sigma-Aldrich)) for 2 h at 4 °C. The supernatant was separated by ultracentrifugation for 30 min, and then incubated with TALON resin (Takara) overnight at 4 °C. The resin was washed with a 15-column volume (CV) wash buffer I (25 mM HEPES, pH 7.4,100 mM NaCl, 10% (v/v) glycerol, 0.05% (w/v) LMNG, 0.01% (w/v) CHS, 20 mM imidazole) and a 15-CV wash buffer II (25 mM HEPES, pH 7.4,100 mM NaCl, 10% (v/v) glycerol, 0.01% (w/v) LMNG, 0.002% (w/v) CHS, 30 mM imidazole). Then elution was carried out with a 3-CV buffer (25 mM HEPES, pH 7.4, 100 mM NaCl, 10% (v/v) glycerol, 0.01% (w/v) LMNG, 0.002% (w/v) CHS, 300 mM imidazole). After the protein was eluted, the imidazole was removed by PD MiniTrap G-25 column (GE Health Care). ET_A_ or ET_B_, miniGα_s/q_, Gβ_1_γ_2_ and Nb35 were mixed at a molar mass ratio of 1:1.2:1.2:1.5, incubated at 24 °C for 1 h; and then 1 μL apyrase (0.5 U/μL) were added, followed by an additional 1-h incubation at 25 °C. The mixture samples were loaded onto the Superdex 200 10/300 column (GE Healthcare) in an equilibration buffer (20 mM HEPES, pH 7.4, 100 mM NaCl, 0.00075% (w/v) LMNG, 0.00015% (w/v) CHS, 0.00025% (w/v) GDN, 100 mM TCEP). The peak fractions containing ET_A_– or ET_B_–miniG_s/q_–β_1_γ_2_–Nb35 complex were collected and concentrated to 2.0–4.0 mg/mL for cryo-EM sample preparation. 10 μM ET-1 or BQ3020 were added to the buffer during the purification process.

### Expression and purification of Fab_301_

As previously reported, Getagozumab^23^ was used to produce the Fab_301_ fragment. The Fab_301_ fragment was codon-optimized and synthesized by GenScript. The corresponding light and heavy chain genes were then subcloned into the pFastBac Dual vector for expression using the Bac-to-Bac baculovirus expression system. Hi5 insect cells were infected with baculovirus at a density of 2 × 10^6^ cells per mL and cultured at 27 °C. Cells were harvested 72 h post infection by centrifugation at 2000 rpm for 30 min, and the clear supernatant was collected. The pH of this supernatant was adjusted to 7.0 before the supernatant was applied to a 2 mL Ni-NTA resin and incubated at 4 °C for 2 h. The column was subsequently washed with a 15-CV buffer containing 20 mM HEPES, pH7.0, 500 mM NaCl, and 20 mM imidazole to remove nonspecifically bound proteins. The protein of interest was eluted from the column using an elution buffer containing 20 mM HEPES, pH 7.0, 100 mM NaCl, and 400 mM imidazole. The eluted protein fractions were then further purified on a Superdex 200 10/300 column, which was equilibrated in a buffer containing 20 mM HEPES, pH7.0, 100 mM NaCl, and 10% glycerol. Monomeric fractions were pooled, concentrated to 8.3 mg/mL, flash-frozen in liquid nitrogen, and stored at –80 °C for future use.

### Fab_301_–ET_A_–anti-BRIL Fab-Nb complex formation and purification

The purification of the ET_A_ receptor was performed analogously to the method described above, with the addition of one of the antagonists — ambrisentan, macitentan, or zibotentan during purification. The anti-Bril Fab was expressed in mammalian cells and purified following the protocol described previously^34^. The anti-Fab Nb was expressed in *E. coli* BL21(DE3) strain and purified according to the previously established methods^35^.

For complex formation, the ET_A_, Fab_301_, anti-BRIL Fab, and anti-Fab Nb were mixed at a molar mass ratio of 1:1.2:1.2:1.5. This mixture was incubated at 4 °C for 4 h, and then concentrated and applied to a Superdex 200 10/300 GL column preequilibrated with the buffer containing 20 mM HEPES, pH 7.4, 100 mM NaCl, 0.00075% (w/v) LMNG, 0.00015% (w/v) CHS, 0.00025% (w/v) GDN, 100 mM TCEP, and 50 μM of the chosen antagonist — either ambrisentan, macitentan or zibotentan. Fractions containing the peak of interest were concentrated to an approximate concentration of 12 mg/mL for cryo-EM specimen preparation.

### Cryo-EM sample preparation and data collection

A total of 3 μL of each complex sample, BQ3020–ET_B_–miniG_s/q_, ET1–ET_B_–miniG_s/q_, ET1–ET_A_–miniG_s/q_, Fab_301_–macitentan–ET_A_, Fab_301_–ambrisentan–ET_A_ or Fab_301_–zibotentan–ET_A_, was applied to glow-discharged 300 mesh alloy grids (CryoMatrix Amorphous alloy film R1.2/1.3), and vitrified by Vitrobot Mark IV (Thermo Fisher Scientific). Excess sample was blotted by a filter paper for 3 s with a blot force of 2 before plunge-freezing in liquid ethane with a FEI Vitrobot Mark IV at 100% humidity and 4 °C. The frozen grids were transferred to liquid nitrogen and stored for data acquisition. Cryo-EM data collection was conducted with the Krios G4 cryo-transmission electron microscope (Thermo Fisher Scientific) operating at 300 kV, equipped with the Falcon 4 Direct Electron Detector (Thermo Fisher Scientific). Movies were recorded at a calibrated magnification of 130,000×, yielding a pixel size of 0.96 Å. A total dose of 60 electrons per square angstrom (e^–^/Å^2^) was administered. Automated data collection was facilitated by the EPU software, utilizing a defocus range spanning from –1.0 μm to –2.0 μm.

### Cryo-EM data processing

The overall cryo-EM data processing workflows for the BQ3020–ET_B_–miniG_s/q_, ET1–ET_B_–miniG_s/q_, ET1–ET_A_–miniG_s/q_, Fab_301_–macitentan–ET_A_, Fab_301_–ambrisentan–ET_A_ and Fab_301_–zibotentan–ET_A_ are shown in Supplementary Figs. S2 and S6. Cryo-EM movie stacks were corrected for beam-induced shifts utilizing the dose-weighting approach in Patch Motion Correction^43^. The contrast transfer function (CTF) parameters were calculated by employing the patch CTF estimation in CryoSPARC^44^.

For the BQ3020–ET_B_–miniG_s/q_–Nb35 complex, a total of 7685 images were imported in CryoSPARC v.4.0.1. A conventional neural network-based method Topaz^45^ implemented in CryoSPARC, was used for particle picking. 2,662,705 particles were extracted and then subjected to iterative 2D classification and ab initio reconstruction. Subsequently, 199,360 particles were selected for heterogeneous refinement. The best class was selected for homogeneous refinement, non-uniform refinement, and local refinement, generating a high-quality density map at a resolution of 3.0 Å. DeepEMhancer was applied to enhance local density. The processing steps for the ET1–ET_B_–miniG_s/q_–Nb35 and ET1–ET_A_–miniG_s/q_–Nb35 complexes, mirrored this approach, with specific details provided in Supplementary Fig. S6.

For Fab_301_–macitentan–ET_A_, Fab_301_–ambrisentan–ET_A_ and Fab_301_–zibotentan–ET_A_ complexes, the initial data processing steps were consistent with those of the G protein complexes. However, additional post-processing strategies were implemented. The Fab_301_–macitentan–ET_A_ complex achieved high-quality density after the standard local refinement; thus, no further optimization was performed. In contrast, for the ambrisentan and zibotentan complexes, masks were generated post local refinement to omit the dynamic regions of Fab_301_. Detailed statistics on the number of images and particles at each processing stage are available in Supplementary Fig. S2.

### Model building and refinement

For Fab_301_–macitentan–ET_A_, Fab_301_–ambrisentan–ET_A_ and Fab_301_–zibotentan–ET_A_ complexes, the AlphaFold2-predicted ET_A_ was used for the receptor modeling. The initial models for BRIL, anti-BRIL Fab and anti-Fab Nb were based on the structures derived from the GPR183 complex structure (PDB: 7TUY)^46^. For the BQ3020–ET_B_–miniG_s/q_–Nb35, ET1–ET_B_–miniG_s/q_–Nb35, and ET1–ET_A_–miniG_s/q_–Nb35 complexes, model building and refinement of the miniG_s/q_ and Nb35 began with the miniG_s/q_ structure from the GPR139–miniG_s/q_ complex structure (PDB: 7VUH)^42^. Model docking into the EM density maps was carried out using Chimera^47^, followed by iterative manual adjustments and rebuilding in Coot^48^. Subsequent refinement was performed using phenix.real_space_refine in PHENIX^49^. Statistical validation of the model was conducted through MolProbity^50^. Structural figures were prepared using ChimeraX^51^. The complete refinement statistics are documented in Supplementary Table S1.

### Intracellular calcium mobilization assay

CHO-K1 cells were cultured in Ham’s F-12K (Kaighn’s) Medium (Gibco-Thermo Fisher Scientific) supplemented with 10% (v/v) fetal bovine serum (FBS; Gibco-Thermo Fisher Scientific), and 100 U/mL Penicillin-Streptomycin (Gibco-Thermo Fisher Scientific) in a humidified incubator at 37 °C with 5% CO_2_. Cells were seeded in 6-cm dishes overnight; when the density reached 60%–80%, the cells were transferred to F12K Medium supplemented with 10% FBS and transfected with 1.5 μg DNA encoding the ET_A_ WT or mutants or 3 μg DNA encoding the ET_B_ WT or mutants using TransIT2020 (Mirus Biosciences). The next day, transfected cells were harvested from the plate using Versene buffer (Gibco-Thermo Fisher Scientific) and seeded into black-sided, clear-bottomed 384-well plates (Agilent) at a density of 1,5000 cells per well. After 20 h, the 1% dFBS medium was removed, and cells were loaded with 20 μL/well of 1× Calcium 6 dye (Molecular Devices) and incubated at 37 °C for 1 h in the dark. 10 μL/well of 3× ET-1 was added and the plates were read using the FLIPR Tetra High Throughput Cellular Screening System (Molecular Devices). To measure antagonist or Fab potency, 10 μL/well of 3× antagonist or Fab was added and incubated with the cells for 30 min at room temperature. The fluorescence intensity was recorded for 2 min after 10 μL/well of 4× ET-1 addition. All data were analyzed using GraphPad Prism 8 and the data are from at least three independent replicate experiments. All plots are shown as means ± SEM. Data were determined using one-way ANOVA followed by Dunnett’s multiple test compared with WT. The top value was normalized to 100% and the bottom value was normalized to 0% for the final presentation. Nonlinear curve fit was performed using a four-parameter logistic equation (log (agonist vs response) or log (inhibitor vs response)).

### MD simulation

MD simulations were performed using the GROMACS2021 software, employing the CHARMM36m force field^52^ and incorporating TIP3 water molecules. Parameters for ambrisentan were derived using the CGenFF force field^53^. To compensate for the missing ICL3 region in the ambrisentan complex structure, a segment from ET_A_ in the G protein complex was used. The complete structure was prepared with the Protein Preparation Wizard in Maestro (2023-1, Schrödinger), which included the determination of the protonation states of residues at pH 7.4 using PROPKA. The protein was embedded into a lipid bilayer composing 150 POPC molecules using CHARMM-GUI^54^, which also facilitated the addition of 0.15 M sodium and chloride ions to neutralize the system’s charge. Energy minimization and equilibration processes were performed following the default protocol of CHARMM-GUI, using a cutoff distance of 12 Å for nonbonded contacts and the Particle Mesh Ewald (PME) method^55^ for long-range van der Waals interactions. Subsequent MD production runs of 500 ns were conducted at a temperature of 310 K and 1 bar using a semi-isotropic Parrinello-Rahman barostat. The final MD trajectories were analyzed and visualized using VMD^56^ software, where the ligand RMSD calculations were also performed.

### Supplementary information

Supplementary Information

## Data Availability

The atomic coordinates for BQ3020–ET_B_–miniG_s/q_, ET1–ET_B_–miniG_s/q_, ET1–ET_A_–miniG_s/q_, Fab_301_–macitentan–ET_A_, Fab_301_–ambrisentan–ET_A_ and Fab_301_–zibotentan–ET_A_ complexes have been deposited in the Protein Data Bank (PDB) with the accession codes 8XVE, 8XVH, 8XVI, 8XVJ, 8XVK and 8XVL, respectively. The EM maps for BQ3020–ET_B_–miniG_s/q_, ET1–ET_B_–miniG_s/q_, ET1–ET_A_–miniG_s/q_, Fab_301_–macitentan–ET_A_, Fab_301_–ambrisentan–ET_A_ and Fab_301_–zibotentan–ET_A_ complexes have been deposited in the Electron Microscopy Data Bank (EMDB) with the codes EMD-38702, EMD-38704, EMD-38705, EMD-38706, EMD-38707 and EMD-38708, respectively.
