# Supplementary material for: Structural basis of antagonist selectivity in endothelin receptors
Source: Cell Discov. 2024 Jul 30;10:79. doi: 10.1038/s41421-024-00705-9 (PMC11286772; doi:10.1038/s41421-024-00705-9)
Supplement: Supplementary file 1 — Supplementary Information [file 41421_2024_705_MOESM1_ESM.pdf]

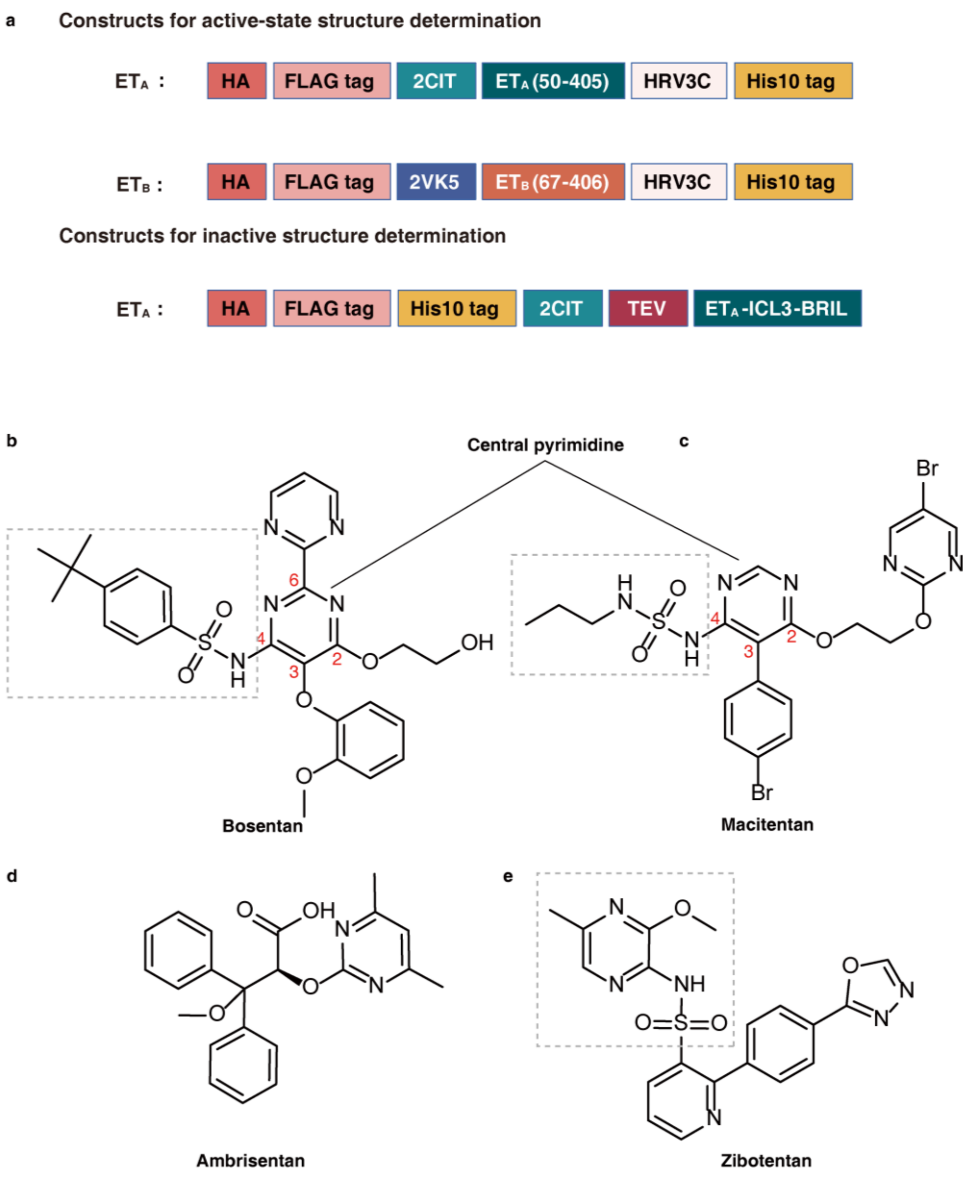

Supplementary Fig. S1 Constructs design for structure determination and chemical structural formulas of macitentan, bosentan, ambisentan, and zibotentan. a, Constructs for active- and inactive-ET$_A$ and active ET$_B$ structural determination. **b-c,** Macitentan retains bosentan's pyrimidine core, but distinguishes itself through a sulfonamide substitution at the fourth position. **d,** Ambrisentan is a propionic acid derivative, featuring a carboxylic acid group in place of the sulfonamide moiety found in macitentan. **e,** Zibotentan is a sulfonamide-based molecule characterized by two bulky substituents attached to the central pyridine ring.

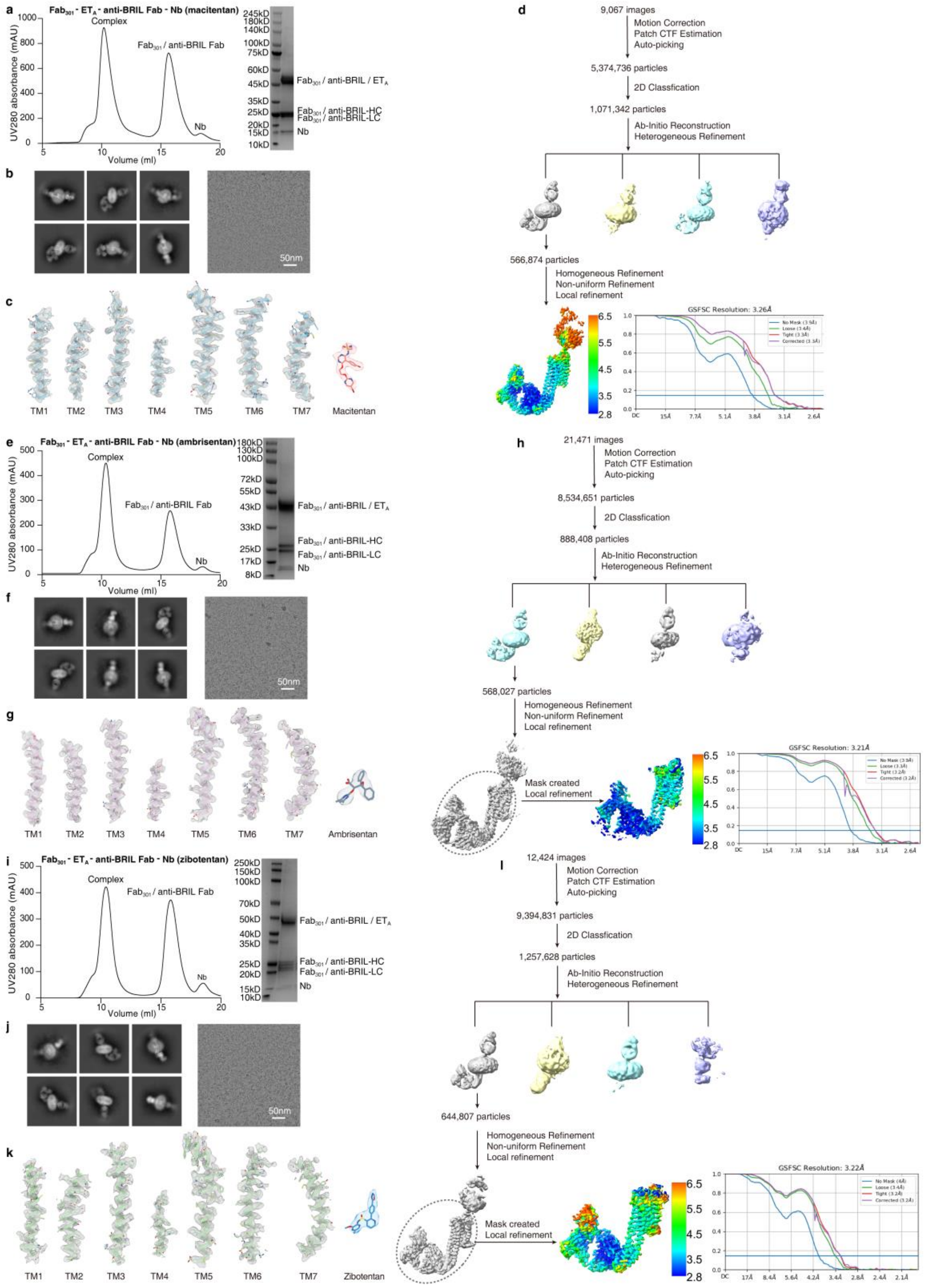

Supplementary Fig. S2 Purification and cryo-EM data processing for macitentan, ambrisentan and zibotentan bound Fab301-ETA-anti-BRIL Fab-Nb complexes. (a, e, i) Representative size exclusion chromatography profiles and SDS–PAGE analysis

of the Fab_{301}-macitentan-ET_A-anti-BRIL Fab-Nb (**a**), Fab_{301}-ambrisentan-ET_A-anti-BRIL Fab-Nb (**e**) and Fab_{301}-zibotentan-ET_A-anti-BRIL Fab-Nb complexes (**i**). **(b, f, j)** Representative cryo-EM images and 2D classification of the Fab_{301}-macitentan-ET_A-anti-BRIL Fab-Nb (**b**), Fab_{301}-ambrisentan-ET_A-anti-BRIL Fab-Nb (**f**) and Fab_{301}-zibotentan-ET_A-anti-BRIL Fab-Nb complexes (**j**). (**c, g, k)** Cryo-EM density maps of each transmembrane helices. (**d, h, l)** Flow chart of the cryo-EM data processing for the Fab_{301}-macitentan-ET_A-anti-BRIL Fab-Nb (**d**), Fab_{301}-ambrisentan-ET_A-anti-BRIL Fab-Nb (**h**) and Fab_{301}-zibotentan-ET_A-anti-BRIL Fab-Nb complexes (**l**). The "Gold-standard" Fourier shell correlation (FSC) curve indicates that the global resolution of the electron density map of the Fab_{301}-macitentan-ET_A-anti-BRIL Fab-Nb complex is 3.1 Å, the Fab_{301}- ambrisentan-ET_A-anti-BRIL Fab-Nb complex is 3.2 Å, and the Fab_{301}-zibotentan-ET_A-anti-BRIL Fab-Nb complex is 3.2 Å.

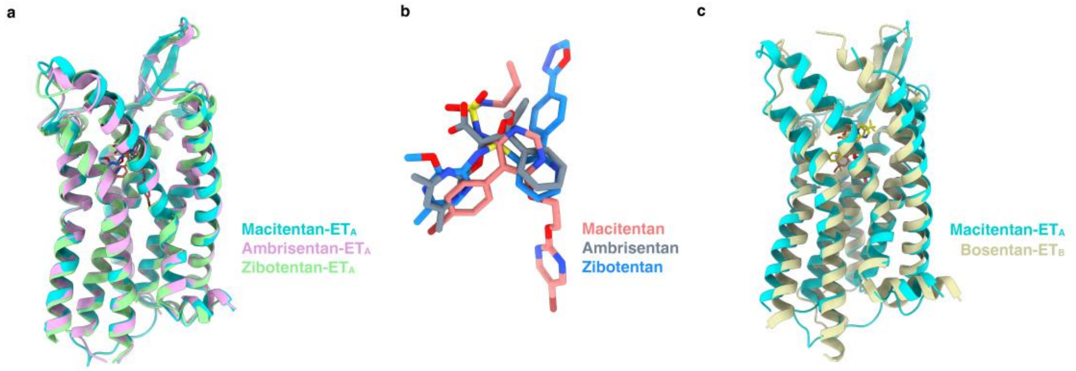

Supplementary Fig. S3 Comparison of the structures of ET_A and ET_B in their inactive states. a, Comparison of the structures of ET_A bound to three different antagonists. **b,** Comparison of the structures of three small molecules. **c,** Structural alignment between macitentan-bound ET_A and bosentan-bound ET_B complex.

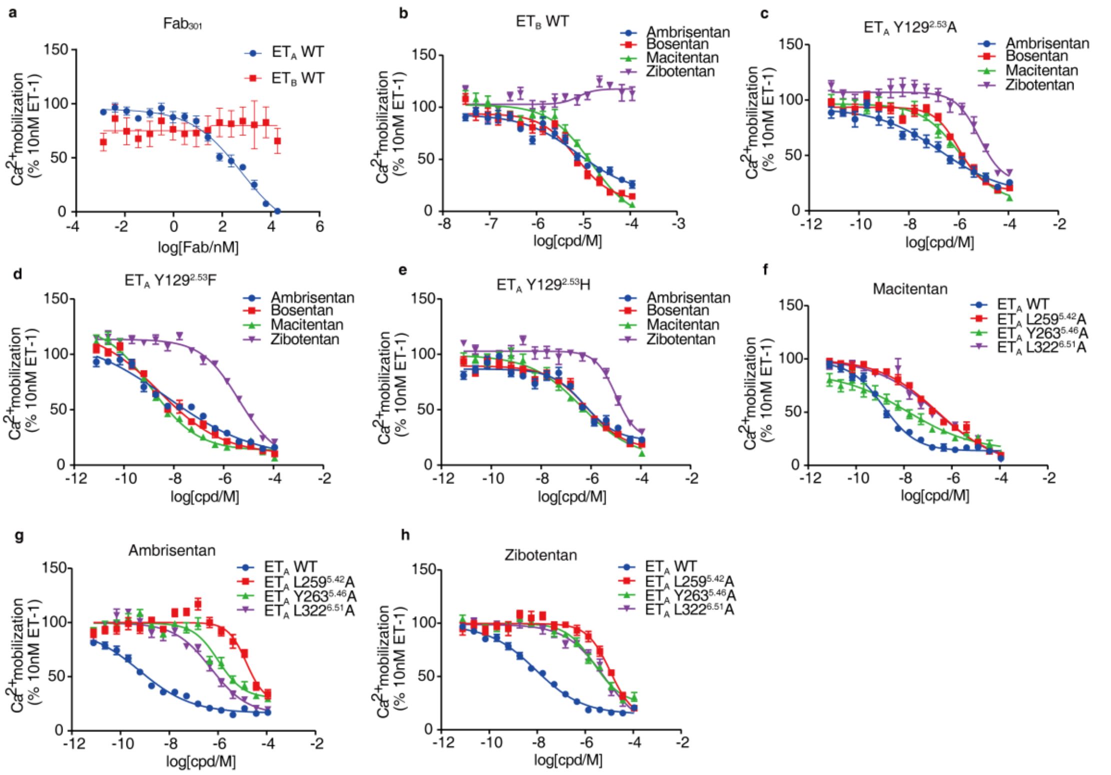

Supplementary Fig. S4 Macitentan, bosentan, ambrisentan, and zibotentan response curves on WT and mutant ET_A/ET_B. a, Fab_{301} antagonism assay for ET_A and ET_B (n =3). **b,** Antagonism assay of four antagonists for ET_B (n = 3). **c-e,** Determination of signals of four antagonists respectively for ET_A Y129$^{2.53}$A (n = 3), Y129$^{2.53}$F (n = 3) and Y129$^{2.53}$H (n = 3). **f-h,** Functional determination of L259$^{5.42}$A (n = 3), Y263$^{5.46}$A (n = 4) and L322$^{6.51}$A (n = 3) for three antagonists. Data are shown as means ± S.E.M. from at least three independent experiments.

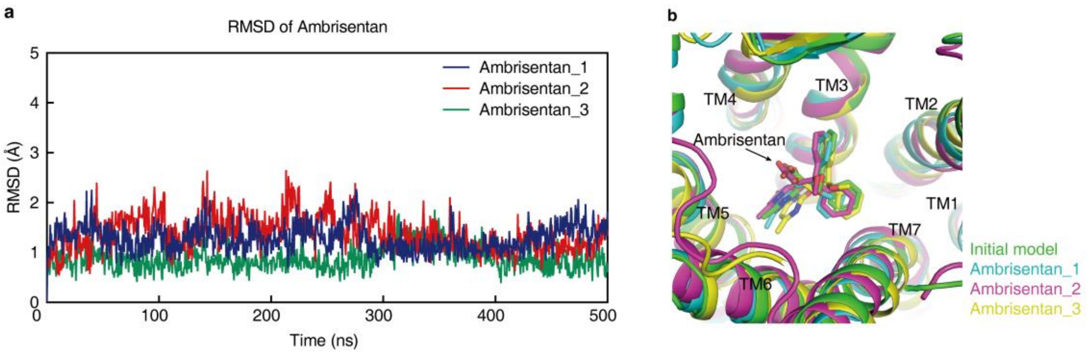

Supplementary Fig. S5 MD simulations of the ambrisentan binding pose in the complex structure. a, The RMSD values of ambrisentan during the MD process. **b,** The comparison between the final frames of the binding poses of ambrisentan in the three MD simulations and the initial binding pose in the complex structure (green sticks).

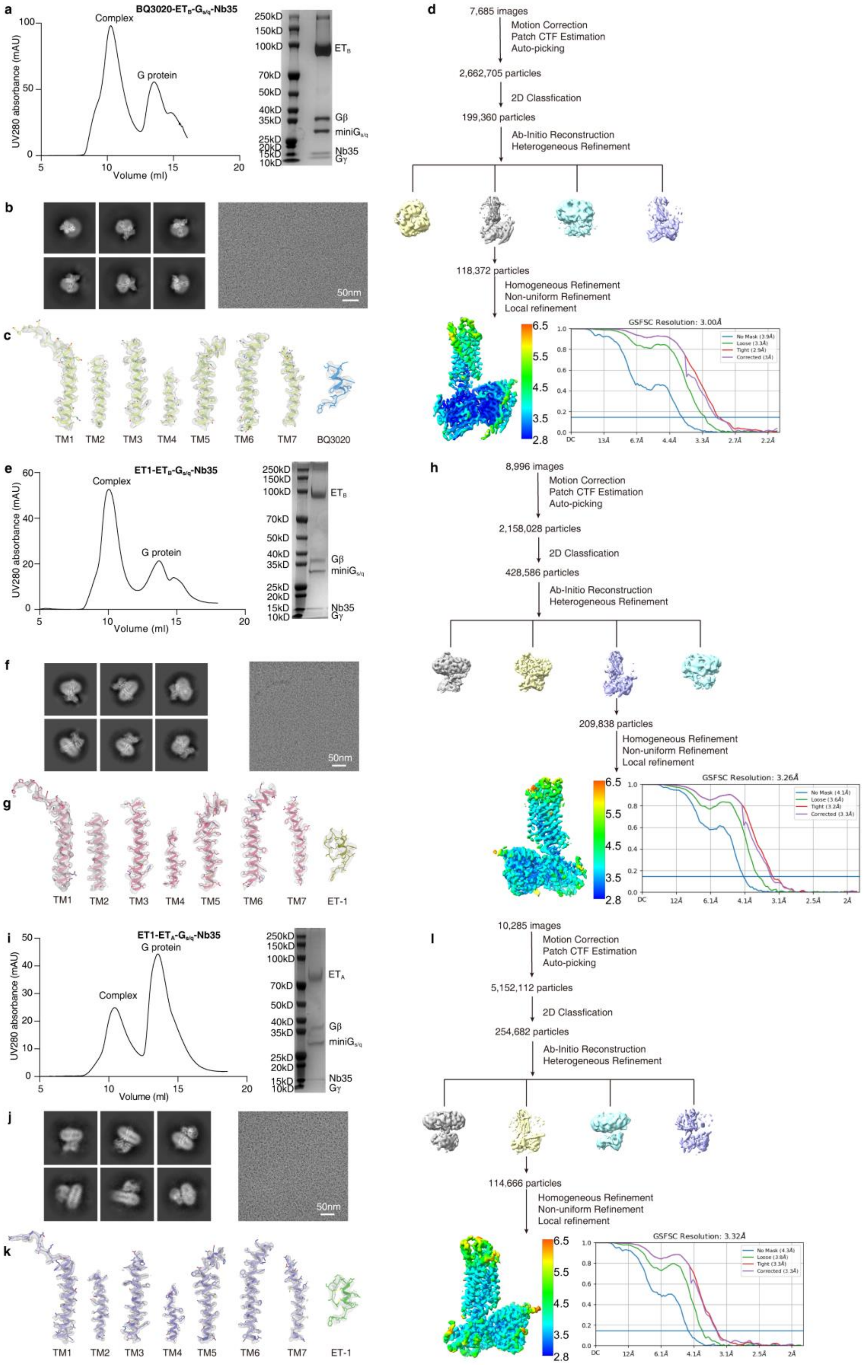

Supplementary Fig. S6 Purification and cryo-EM data processing for BQ3020-

ET_B-miniG$_{s/q}$-Nb35, ET1-ET_B-miniG$_{s/q}$-Nb35, and ET1-ET_A-miniG$_{s/q}$-Nb35 complexes. a, e, i, Representative size exclusion chromatography profiles and SDS–PAGE analysis of the BQ3020-ET_B-miniG$_{s/q}$-Nb35 (**a**), ET1-ET_B-miniG$_{s/q}$-Nb35 (**e**) and ET1-ET_A-miniG$_{s/q}$-Nb35 complexes (**i**). **b, f, j,** Representative cryo-EM images and 2D classification of the BQ3020-ET_B-miniG$_{s/q}$-Nb35 (**b**), ET1-ET_B-miniG$_{s/q}$-Nb35 (**f**) and ET1-ET_A-miniG$_{s/q}$-Nb35 complexes (**j**). **c, g, k,** Cryo-EM density maps of each transmembrane helices. **d, h, l,** Flow chart of the cryo-EM data processing for the BQ3020-ET_B-miniG$_{s/q}$-Nb35 (**d**), ET1-ET_B-miniG$_{s/q}$-Nb35 (**h**) and ET1-ET_A-miniG$_{s/q}$-Nb35 complexes (**l**). The "Gold-standard" Fourier shell correlation (FSC) curve indicates that the global resolution of the electron density map of the BQ3020-ET_B-miniG$_{s/q}$-Nb35 complex is 3.0 Å, the ET1-ET_B-miniG$_{s/q}$-Nb35 complex is 3.2 Å, and the ET1-ET_A-miniG$_{s/q}$-Nb35 complex is 3.3 Å.

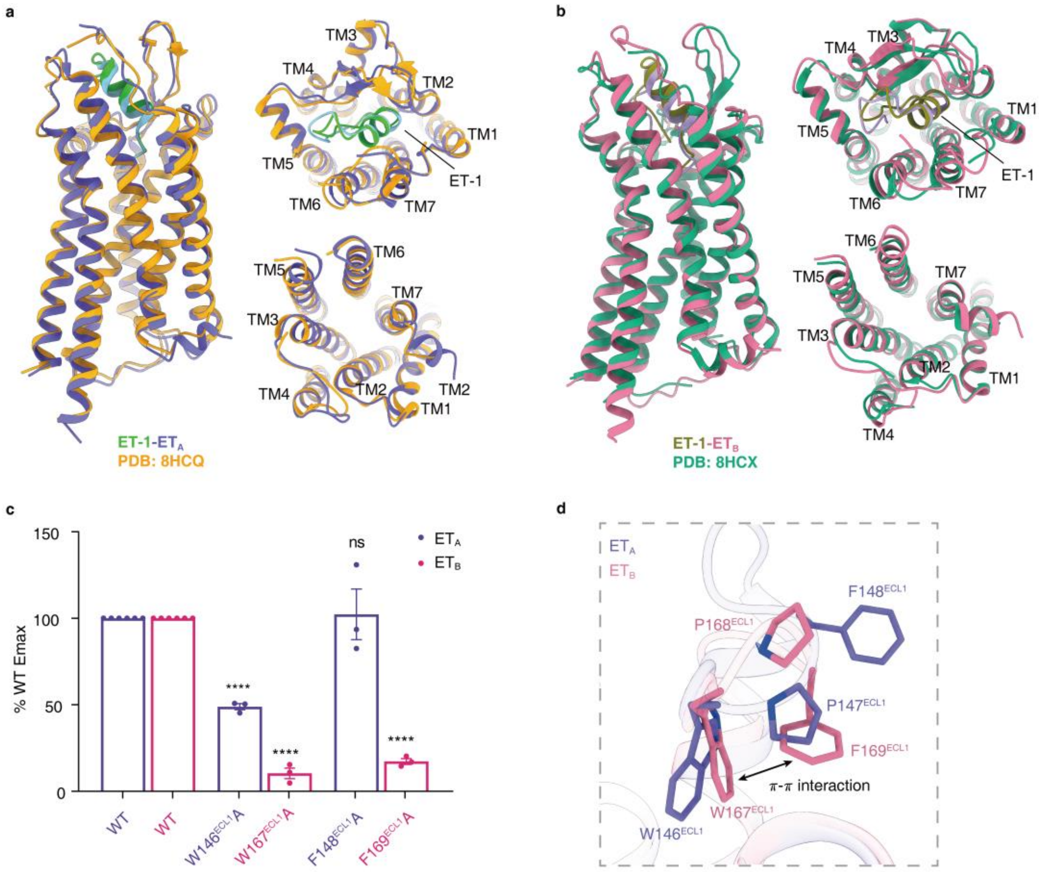

Supplementary Fig. S7 Structural comparison of ET-1 bound active ET$_A$ and ET$_B$ with previously reported ETRs structures. Compared with the previously reported conformations, the G protein-coupled complexes of ET-1 with ET$_A$ **(a)** and ET$_B$ **(b)** have similar conformations, and ET-1 adopts a comparable binding pose in both ETRs. **c,** Response of ET-1 on WT and mutants of ET$_A$ and ET$_B$. **d,** ECL1 conformational comparison between ET$_A$ and ET$_B$, the bidirectional arrow indicates π-π interactions between F169^{ECL1} and W167^{ECL1}.

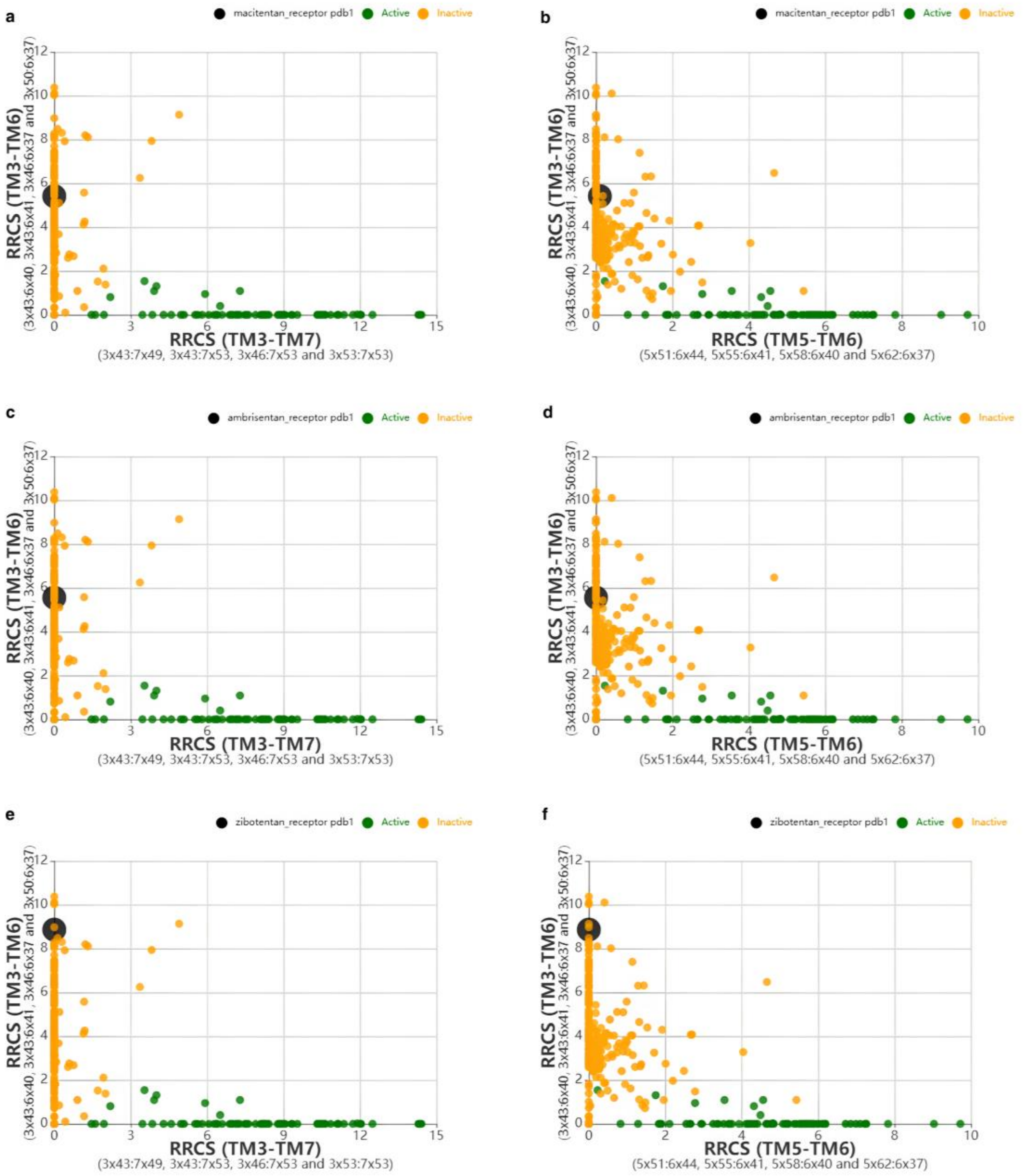

Supplementary Fig. S8 RRCS score analysis of three antagonists bound ET_A structures. The Residue-Residue Contact Score (RRCS) tool was used to confirm that residue contacts in antagonist-bound ET_A feature the characteristics of inactive-state class A GPCRs. The calculation of RRCS was performed using the following website http://gpcranalysis.com/#/.

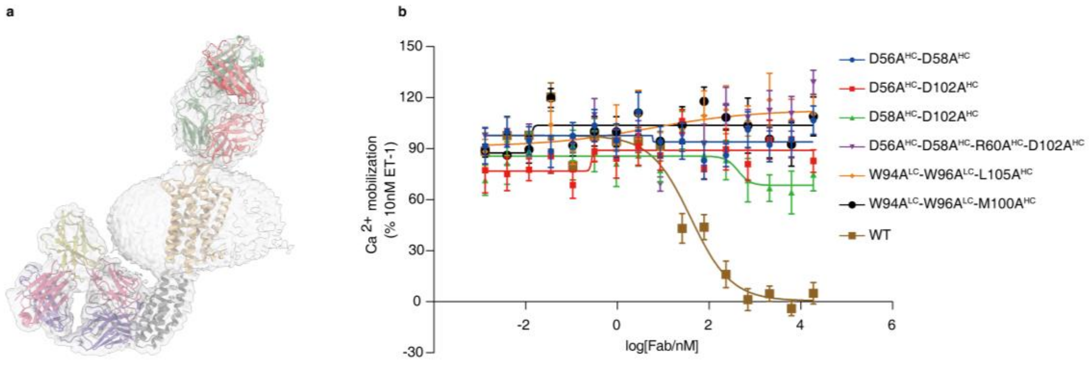

Supplementary Fig. S9 The structure and mutational functional assay of Fab$_{301}$. a, Alignment of density with corresponding AF2 predicted structure (receptor and the associated Fab$_{301}$ portion). **b,** Antagonistic signaling experiment of Fab$_{301}$ mutants with ET$_{A}$.

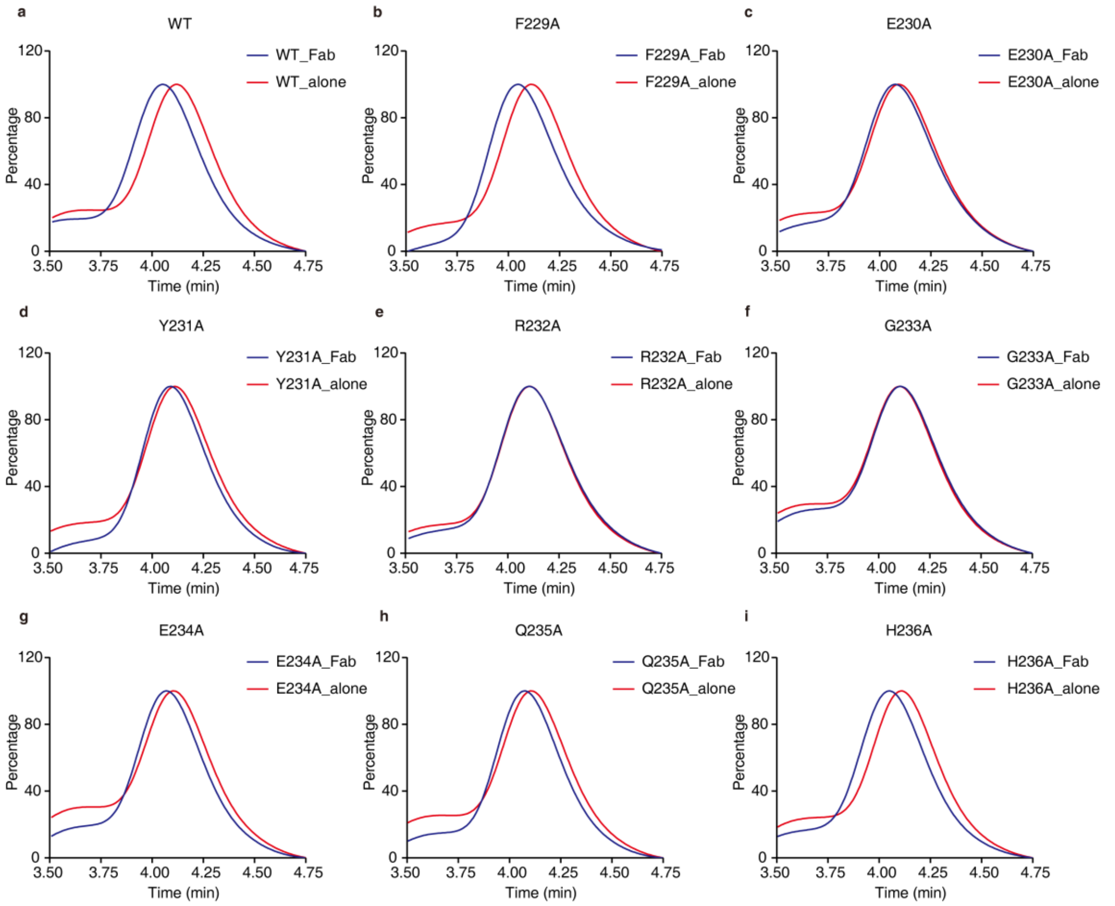

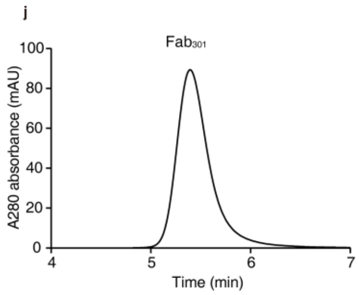

k

	Receptor_alone RetTime (min)	Receptor_Fab RetTime (min)	Δt (time)
WT	4.120	4.051	0.069
F229A	4.110	4.047	0.063
E230A	4.091	4.077	0.014
Y231A	4.107	4.090	0.017
R232A	4.109	4.105	0.004
G233A	4.104	4.107	-0.003
E234A	4.105	4.070	0.035
Q235A	4.110	4.074	0.036
H236A	4.107	4.050	0.057

Supplementary Fig. S10 SEC curves of mutants-alone, mutants-Fab301 complex and Fab301-alone. a-i, Comparison of SEC curves for each mutant and the current mutants combined with Fab. For data from the same comparison group, the protein absorbance (280 nm) was normalized, with the maximum value defined as 100% and the minimum value defined as 0%. **j,** SEC curve of Fab301. **k,** SEC retention times for mutants and mutants-Fab301 complex, Δt represents $T_{mutants}$-$T_{complex}$.

Supplementary Table S1 Cryo-EM data collection, model refinement, and validation statistics.

	ET_A-Macitentan	ET_A-Ambrisentan	ET_A-Zibotentan	$G_{s/q}$-ET_B-BQ3020	$G_{s/q}$-ET_B-ET1	$G_{s/q}$-ET_A-ET1
**Data collection and processing**						
Magnification	130,000	130,000	130,000	130,000	130,000	130,000
Voltage (kv)	300	300	300	300	300	300
Electron exposure (e-/Å^2)	60	60	60	60	60	60
Defocus range (μm)	-1.0~-2.0	-1.0~-2.0	-1.0~-2.0	-1.0~-2.0	-1.0~-2.0	-1.0~-2.0
images	9067	21471	12424	7685	8996	10285
Pixel size (Å)	0.96	0.96	0.96	0.96	0.96	0.96
Symmetry imposed	C1	C1	C1	C1	C1	C1
Final particles	566,874	568,027	644,807	118,372	209,838	114,666
Map resolution	3.26	3.21	3.22	3.00	3.26	3.32
FSC threshold	0.143	0.143	0.143	0.143	0.143	0.143
**Refinement**						
Initial model used (PDB code)	7TUY	7TUY	7TUY	7VUH	7VUH	7VUH
Map sharping *B* factor (Å^2)	-98.2	-122.4	-103.7	-108.5	-125.7	-123.5
CC of map-model (mask)	0.73	0.72	0.71	0.78	0.73	0.72
Model composition						
Non-hydrogen atoms	7431	7388	7337	8524	8544	8587
Protein residues	954	949	942	1078	1080	1084
Ligand	1	1	1	0	0	0
B-factors						
Protein	64.15	80.27	64.99	73.52	81.19	86.14
Ligand	48.20	64.34	32.83	0	0	0
R.M.S. deviations						
Bond lengths (Å)	0.003	0.004	0.005	0.004	0.002	0.004
Bond angles (˚)	0.667	0.682	1.038	0.627	0.574	0.621
Validation						
MolProbity score	1.83	1.96	1.46	1.43	1.43	1.87
Clash score	9.43	11.26	9.89	7.87	6.45	9.83
Poor rotamers (%)	0.49	0.00	0.25	0.65	0.21	0.43
Ramachandran plot						

Favored (%)	95.23	94.13	94.30	98.02	97.65	95.04
Allowed (%)	4.77	5.87	5.70	1.98	2.35	4.96
Disallowed (%)	0.00	0.00	0.00	0.00	0.00	0.00
EMD code	EMD-38706	EMD-38707	EMD-38708	EMD-38702	EMD-38704	EMD-38705
PDB code	8XVJ	8XVK	8XVL	8XVE	8XVH	8XVI

Supplementary information, Table S2 Effects of four antagonists on ET_A/ET_B mutants. Calcium mobilization assay was performed to evaluate the potency of bosentan, macitentan, ambrisentan, and zibotentan on ET_A/ET_B mutants. The surface expression of each ET_A/ET_B mutant was normalized to wild-type (WT) receptor, which was set to 100%. Data are presented as means ± S.E.M. from at least three independent experiments in triplicate (n ≥ 3). Statistical significance was assessed by one-way ANOVA followed by Dunnett's post-test, comparing the response with that of the WT: $^{*}P<0.05$, $^{**}P<0.01$, $^{***}P<0.001$, and $^{****}P<0.0001$. ND, not detectable.

Receptors	Constructs	pIC_{50}±S.E.M. (Ambrisentan)	pIC_{50}±S.E.M. (Macitentan)	pIC_{50}±S.E.M. (Bosentan)	pIC_{50}±S.E.M. (Zibotentan)	Surface expression (% of WT)
ET_A	WT	9.41±0.16	8.93±0.08	7.94±0.22	7.87±0.22	100±0
	$Y129^{2.53}A$	$6.69±0.41^{****}$	$5.99±0.23^{****}$	$5.98±0.08^{****}$	$5.20±0.09^{****}$	97.39±9.97
	$Y129^{2.53}F$	8.63±0.52	8.92±0.07	8.59±0.03	$5.37±0.02^{****}$	108.10±10.29
	$Y129^{2.53}H$	$6.24±0.01^{****}$	$6.05±0.35^{****}$	$6.09±0.11^{****}$	$5.02±0.08^{****}$	102.10±3.98
	$F161^{3.28}A$	$7.05±0.26^{****}$	$7.75±0.18^{***}$	$6.42±0.06^{****}$	$5.26±0.09^{****}$	109.10±8.96
	$F161^{3.28}V$	ND	ND	ND	ND	110.30±11.40
	$L259^{5.42}A$	$4.81±0.04^{****}$	$6.63±0.06^{****}$	$4.95±0.13^{****}$	$4.99±0.03^{****}$	100.00±5.86
	$Y263^{5.46}A$	$6.09±0.05^{****}$	$7.81±0.40^{**}$	$5.59±0.13^{****}$	$5.59±0.09^{****}$	96.56±12.95
	$L322^{6.51}A$	$6.24±0.11^{****}$	$6.46±0.32^{****}$	8.11±0.16	$5.22±0.21^{****}$	81.93±9.97
	$R326^{6.55}A$	$5.10±0.05^{****}$	$7.44±0.21^{***}$	$4.33±0.45^{****}$	$5.39±0.05^{****}$	109.50±5.22
ET_B	WT	5.03±0.22	4.64±0.12	5.22±0.06	ND	100±0
	$H150^{2.53}Y$	$6.32±0.28^{**}$	$7.87±0.04^{****}$	$6.98±0.17^{****}$	4.25±0.20	89.94±5.25
	$V177^{3.28}F$	$9.16±0.15^{****}$	4.83±0.12	$5.79±0.08^{*}$	ND	82.15±3.63
	$V177^{3.28}F+H150^{2.53}Y$	$9.27±0.17^{****}$	$8.36±0.19^{****}$	$7.98±0.20^{****}$	4.48±0.13	93.15±5.82

Supplementary information, Table S3 Effects of ET-1 on ET_A/ET_B mutants. Calcium mobilization assay was performed to evaluate the efficacy and potency of ET-1 on ET_A or ET_B. The surface expression of each ET_A or ET_B mutant was normalized to wild-type (WT) receptor, which was set to 100%. Data are presented as means ± S.E.M. from at least three independent experiments in triplicate ($n \geq 3$). Statistical significance was assessed by one-way ANOVA followed by Dunnett's post-test, comparing the response with that of the WT: $^{*}P<0.05$, $^{**}P<0.01$, $^{***}P<0.001$, and $^{****}P<0.0001$. ND, not detectable.

Receptors	Constructs	pEC_{50}± S.E.M.	E_{max} (% of WT)	Span (% of WT)	Surface expression (% of WT)
ET_A	WT	8.70±0.06	100±0	100±0	100±0
	$W146^{ECL1}A$	8.71±0.14	$48.89±1.76^{****}$	$49.48±4.28^{****}$	77.74±6.64
	$F148^{ECL1}A$	8.26±0.15	102.30±14.65	95.26±12.41	84.82±13.68
	$E230^{ECL2}A$	8.76±0.14	$62.87±7.17^{***}$	$59.25±4.55^{****}$	106.50±6.48
	$Y231^{ECL2}A$	$7.72±0.14^{****}$	$33.48±5.51^{****}$	$29.69±4.82^{****}$	98.95±4.66
	$R232^{ECL2}A$	8.54±0.06	$65.27±3.56^{***}$	$64.62±2.58^{****}$	99.48±8.30
	$G233^{ECL2}A$	8.69±0.11	82.17±3.18	$78.17±1.69^{**}$	112.60±11.19
	$E234^{ECL2}A$	8.98±0.06	86.54±5.76	$79.95±4.41^{**}$	75.60±10.29
	$Q235^{ECL2}A$	8.89±0.06	$77.88±3.40^{*}$	$71.29±2.46^{****}$	90.02±10.05
ET_B	WT	8.73±0.09	100±0	100±0	100±0
	$W167^{ECL1}A$	8.36±0.38	$10.38±3.07^{****}$	$14.22±1.25^{****}$	80.78±3.75
	$W169^{ECL1}A$	ND	$17.25±1.67^{****}$	$11.44±3.08^{****}$	80.41±12.96
